# Crystal structure of 4-chloro-2-iodo­aniline

**DOI:** 10.1107/S1600536814016869

**Published:** 2014-08-01

**Authors:** Taylor R. Quinn, Joseph M. Tanski

**Affiliations:** a Department of Chemistry, Vassar College, Poughkeepsie, NY 12604, USA

**Keywords:** crystal structure, halogen–halogen inter­action, aniline, π-stacking

## Abstract

In the crystal structure of the title compound, C_6_H_5_ClIN, the amino group engages in N—H⋯N hydrogen bonding, creating [100] chains. A Cl⋯I contact is observed [3.7850 (16) Å]. The parallel planes of neigbouring mol­ecules reveal highly offset π-stacking characterized by a centroid–centroid distance of 4.154 (1), a centroid-to-plane distance of 3.553 (3) and ring-offset slippage of 2.151 (6) Å.

## Related literature   

For the synthesis and vibrational spectroscopic analysis of 4-chloro-2-iodo­aniline, see: Hoque *et al.* (2013 ▶). For the dehalo­genation of dihalogenated anilines in human liver microsomes, see: Zhang *et al.* (2011 ▶). For the crystal structures of related monohalogenated anilines, see: Trotter *et al.* (1966 ▶); Parkin *et al.* (2005 ▶) and of dihalogenated anilines, see: Xu *et al.* (2008 ▶). For halogen–halogen inter­actions, see: Pedireddi *et al.* (1994 ▶) and for π-stacking, see: Lueckheide *et al.* (2013 ▶). For van der Waals radii, see: Bondi (1964 ▶).
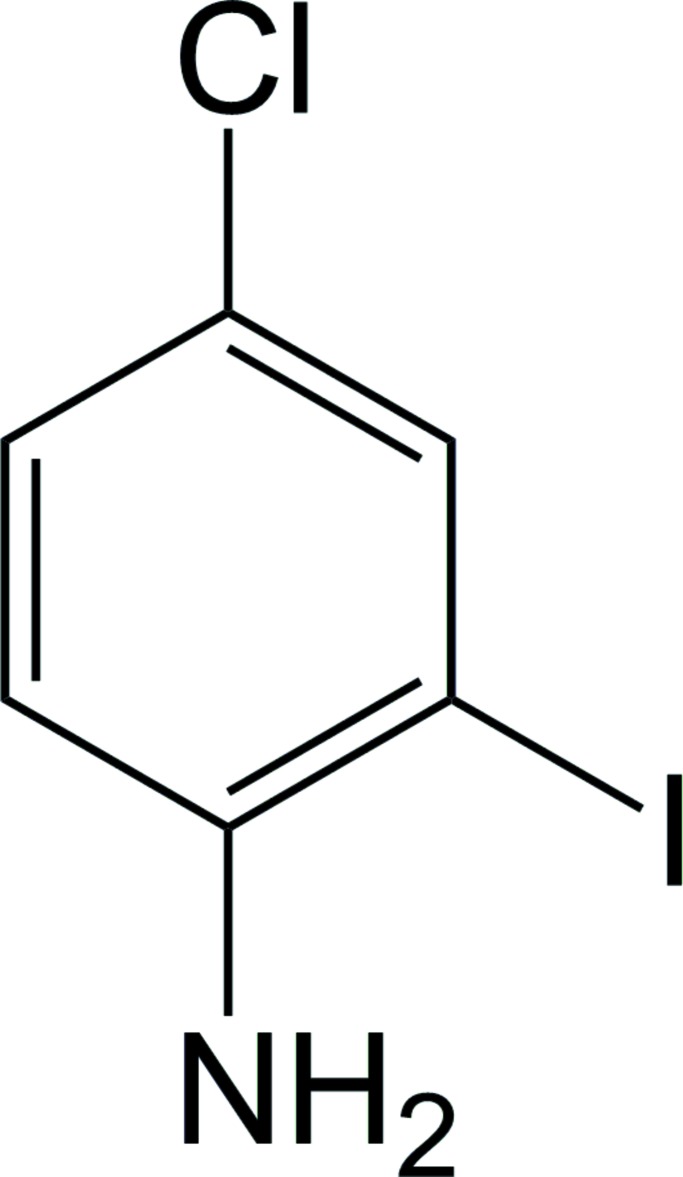


## Experimental   

### Crystal data  

C_6_H_5_ClIN
*M* *_r_* = 253.46
Orthorhombic, 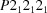

*a* = 4.1538 (4) Å
*b* = 11.3685 (11) Å
*c* = 15.8550 (16) Å
*V* = 748.71 (13) Å^3^
*Z* = 4
Mo *K*α radiation
μ = 4.54 mm^−1^
*T* = 125 K
0.20 × 0.10 × 0.05 mm

### Data collection  

Bruker APEXII CCD diffractometer
Absorption correction: multi-scan (*SADABS*; Bruker, 2007 ▶) *T*_min_ = 0.56, *T*_max_ = 0.81
11850 measured reflections
2281 independent reflections
2007 reflections with *I* > 2σ(*I*)
*R*_int_ = 0.066

### Refinement  

*R*[*F* ^2^ > 2σ(*F* ^2^)] = 0.029
*wR*(*F* ^2^) = 0.053
*S* = 1.02
2281 reflections
88 parameters
2 restraints
H atoms treated by a mixture of independent and constrained refinement
Δρ_max_ = 0.96 e Å^−3^
Δρ_min_ = −1.03 e Å^−3^
Absolute structure: Flack *x* determined using 742 quotients [(*I* ^+^)−(*I* ^−^)]/[(*I* ^+^)+(*I* ^−^)] (Parsons *et al.*, 2013 ▶)
Absolute structure parameter: −0.03 (3)

### 

Data collection: *APEX2* (Bruker, 2007 ▶); cell refinement: *SAINT* (Bruker, 2007 ▶); data reduction: *SAINT*; program(s) used to solve structure: *SHELXS97* (Sheldrick, 2008 ▶); program(s) used to refine structure: *SHELXL2014* (Sheldrick, 2008 ▶); molecular graphics: *SHELXTL2014* (Sheldrick, 2008 ▶); software used to prepare material for publication: *SHELXTL2014*, *OLEX2* (Dolomanov *et al.*, 2009 ▶) and *Mercury* (Macrae *et al.*, 2006 ▶).

## Supplementary Material

Crystal structure: contains datablock(s) global, I. DOI: 10.1107/S1600536814016869/jj2191sup1.cif

Structure factors: contains datablock(s) I. DOI: 10.1107/S1600536814016869/jj2191Isup2.hkl

Supporting information file. DOI: 10.1107/S1600536814016869/jj2191Isup3.cml

**Fig. 1.**
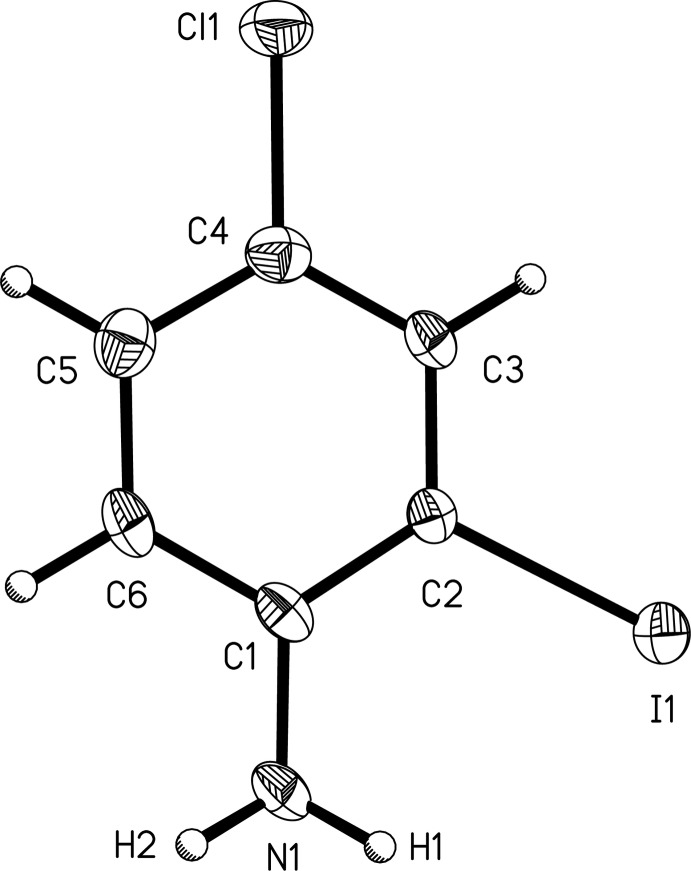
A view of title compound, with atom numbering scheme. Displacement ellipsoids are shown at the 50% probability level.

**Fig. 2.**
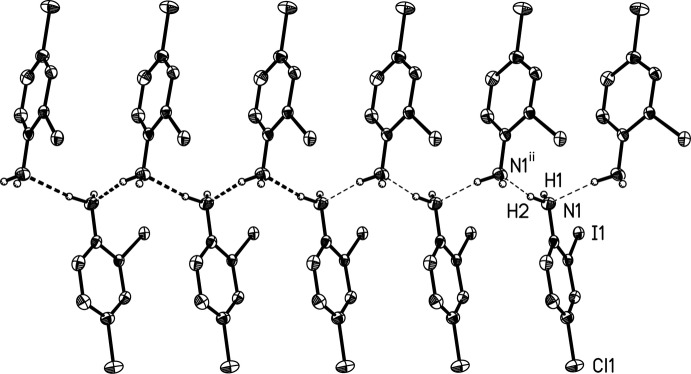
A view of the hydrogen bonding in 4-Chloro-2-iodoaniline forming a chain parallel to the crystallographic a-axis. Displacement ellipsoids are shown at the 50% probability level; hydrogen atoms on carbon removed for clarity. For symmetry code (ii), see Table 1.

**Fig. 3.**
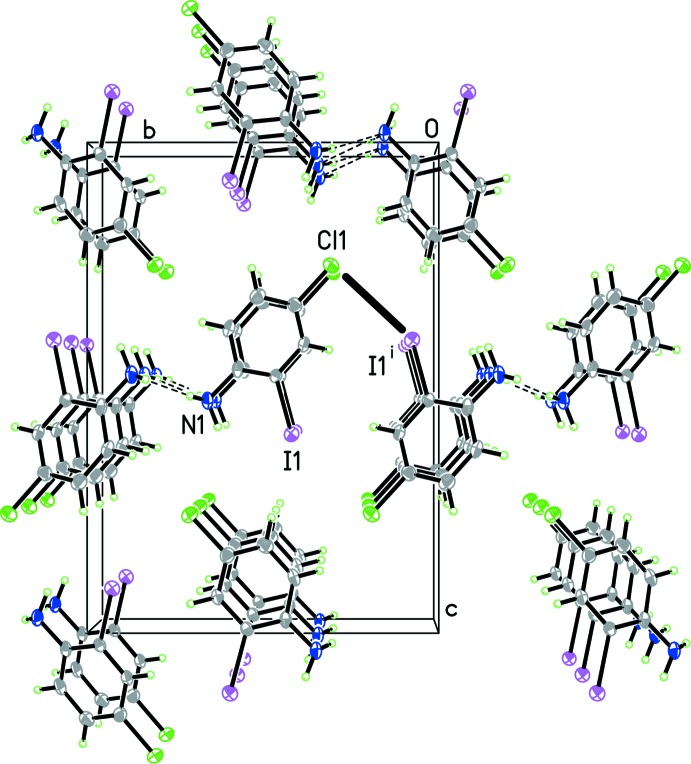
A view of the offset face-to-face π-stacking and Cl···Ii contact (thick solid line) in the packing of 4-Chloro-2-iodoaniline. Displacement ellipsoids are shown at the 50% probability level. Symmetry code (i): x - 1/2, -y + 3/2, -z.

. DOI: 10.1107/S1600536814016869/jj2191fig1.tif
A view of title compound, with atom numbering scheme. Displacement ellipsoids are shown at the 50% probability level.

a . DOI: 10.1107/S1600536814016869/jj2191fig2.tif
A view of the hydrogen bonding in 4-Chloro-2-iodo­aniline forming a chain parallel to the crystallographic *a*-axis. Displacement ellipsoids are shown at the 50% probability level; hydrogen atoms on carbon removed for clarity. For symmetry code (ii), see Table 1.

i x y z . DOI: 10.1107/S1600536814016869/jj2191fig3.tif
A view of the offset face-to-face π-stacking and Cl⋯I^i^ contact (thick solid line) in the packing of 4-Chloro-2-iodo­aniline. Displacement ellipsoids are shown at the 50% probability level. Symmetry code: (i): *x* − 1/2, −*y* + 3/2, −*z*.

CCDC reference: 1015344

Additional supporting information:  crystallographic information; 3D view; checkCIF report

## Figures and Tables

**Table 1:** Hydrogen-bond geometry (Å, °)

*D*—H⋯*A*	*D*—H	H⋯*A*	*D*⋯*A*	*D*—H⋯*A*
N1—H2⋯N1^ii^	0.90 (2)	2.28 (3)	3.142 (6)	161 (5)

Symmetry code: (ii) 
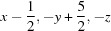
.

## supplementary crystallographic information

### S1. Structural commentary

Dihalogenated anilines such as the title compound exhibit different toxicities depending on the identity, number and substitution pattern of the halogens on the aniline ring, and the mechanism of halogen activiation in differently substituted dihalogenated anilines by gluta­thione has been studied using human liver microsomes (Zhang *et al.*, 2011). The title compound may be synthesized using selective *ortho*-iodination of 4-chloro­aniline (Hoque *et al.*, 2013). The C—Cl and C—I bond lengths of 1.755 (6) Å and 2.101 (5) Å in the title compound (Fig. 1) are similar to those found in the corresponding mono-substituted anilines, 4-chloro­aniline with C—Cl bond length 1.75 Å (Trotter *et al.*, 1966) and 2-iodo­aniline with C—I bond length 2.103 (7) Å (Parkin *et al.*, 2005). Further, the C—Cl and C—I bond lengths are similar to those found in the isomer where the positions of the halides are reversed, 2-chloro-4-iodo­aniline, with C—Cl bond length 1.742 (4) Å and C—I bond length 2.103 (4) Å (Xu *et al.*, 2008).

In the structure of the titular compound, cooperative inter­molecular hydrogen bonding with one of the two amine protons, H2, links the molecules into a one-dimensional chain running down the crystallographic *a*-axis (Fig. 2, Table 1). The other amine proton, H1, does not engage in any significant hydrogen bonding inter­action. There is also an inter­molecular halogen-halogen inter­action between chlorine and iodine, with a Cl···I^i^ distance of 3.7850 (16) Å (Fig. 3) which is slightly longer than the sum of the van der Waals radii of chlorine and iodine, 3.73 Å (Bondi, 1964) [symmetry code (i): x - 1/2, -y + 3/2, -z]. For a discussion of halogen···halogen inter­actions, see Pedireddi *et al.*, 1994. The parallel planes of neigboring aromatic molecules reveal a highly offset face-to-face π-stacking (Fig. 3) characterized by a ring centroid-to-centroid distance of 4.154 (1) Å, centroid-to-plane distance of 3.553 (3) Å, and ring-offset slippage parameter of 2.151 (6) Å (Lueckheide *et al.*, 2013).

### S2. Synthesis and crystallization

Crystalline 4-Chloro-2-iodo­aniline (I) was purchased from Aldrich Chemical Company, USA.

### S3. Refinement

Crystal data, data collection and structure refinement details are summarized in Table 1. All non-hydrogen atoms were refined anisotropically. Hydrogen atoms on carbon were included in calculated positions and refined using a riding model at C–H = 0.95 Å and *U*_iso_(H) = 1.2 × *U*_eq_(C) of the aryl C-atoms. The hydrogen atoms on nitro­gen were located in the difference map and refined semifreely with the help of a distance restraint, d(N—H) 0.90 (2) Å and *U*_iso_(H) = 1.2 × *U*_eq_(N). The extinction parameter (EXTI) refined to zero and was removed from the refinement.

### Crystal data

**Table:**

C_6_H_5_ClIN	*F*(000) = 472
*M**_r_* = 253.46	*D*_x_ = 2.249 Mg m^−^^3^
Orthorhombic, *P*2_1_2_1_2_1_	Mo *K*α radiation, λ = 0.71073 Å
Hall symbol: P 2ac 2ab	Cell parameters from 5580 reflections
*a* = 4.1538 (4) Å	θ = 2.2–30.3°
*b* = 11.3685 (11) Å	µ = 4.54 mm^−^^1^
*c* = 15.8550 (16) Å	*T* = 125 K
*V* = 748.71 (13) Å^3^	Plate, colourless
*Z* = 4	0.20 × 0.10 × 0.05 mm

### Data collection

**Table:**

Bruker APEXII CCD diffractometer	2281 independent reflections
Radiation source: fine-focus sealed tube	2007 reflections with *I* > 2σ(*I*)
Graphite monochromator	*R*_int_ = 0.066
Detector resolution: 8.3333 pixels mm^-1^	θ_max_ = 30.5°, θ_min_ = 2.2°
φ and ω scans	*h* = −5→5
Absorption correction: multi-scan (*SADABS*; Bruker, 2007)	*k* = −16→16
*T*_min_ = 0.56, *T*_max_ = 0.81	*l* = −22→22
11850 measured reflections	

### Refinement

**Table:**

Refinement on *F*^2^	Secondary atom site location: difference Fourier map
Least-squares matrix: full	Hydrogen site location: mixed
*R*[*F*^2^ > 2σ(*F*^2^)] = 0.029	H atoms treated by a mixture of independent and constrained refinement
*wR*(*F*^2^) = 0.053	*w* = 1/[σ^2^(*F*_o_^2^) + (0.0156*P*)^2^] where *P* = (*F*_o_^2^ + 2*F*_c_^2^)/3
*S* = 1.02	(Δ/σ)_max_ = 0.001
2281 reflections	Δρ_max_ = 0.96 e Å^−^^3^
88 parameters	Δρ_min_ = −1.03 e Å^−^^3^
2 restraints	Absolute structure: Flack x determined using 742 quotients [(*I*^+^)-(*I*^-^)]/[(*I*^+^)+(*I*^-^)] (Parsons *et al.*, 2013)
Primary atom site location: structure-invariant direct methods	Absolute structure parameter: −0.03 (3)

### Special details

**Table:**

Geometry. All e.s.d.'s (except the e.s.d. in the dihedral angle between two l.s. planes) are estimated using the full covariance matrix. The cell e.s.d.'s are taken into account individually in the estimation of e.s.d.'s in distances, angles and torsion angles; correlations between e.s.d.'s in cell parameters are only used when they are defined by crystal symmetry. An approximate (isotropic) treatment of cell e.s.d.'s is used for estimating e.s.d.'s involving l.s. planes.
Refinement. Refined as a 2-component inversion twin.

### Fractional atomic coordinates and isotropic or equivalent isotropic displacement parameters (Å^2^)

**Table:**

	*x*	*y*	*z*	*U*_iso_*/*U*_eq_	
I1	1.19309 (8)	0.92075 (3)	−0.09588 (2)	0.02161 (9)	
Cl1	1.0100 (4)	0.80636 (12)	0.24943 (10)	0.0322 (3)	
N1	0.8012 (13)	1.1528 (4)	−0.0258 (3)	0.0236 (9)	
H1	0.795 (14)	1.124 (5)	−0.0783 (19)	0.028*	
H2	0.625 (10)	1.196 (4)	−0.017 (4)	0.028*	
C1	0.8463 (11)	1.0685 (4)	0.0375 (3)	0.0187 (10)	
C2	1.0139 (12)	0.9638 (4)	0.0242 (3)	0.0167 (10)	
C3	1.0695 (12)	0.8840 (4)	0.0886 (3)	0.0190 (10)	
H3	1.1867	0.8136	0.0784	0.023*	
C4	0.9513 (13)	0.9087 (5)	0.1680 (3)	0.0233 (11)	
C5	0.7803 (14)	1.0111 (5)	0.1839 (4)	0.0255 (12)	
H5	0.6977	1.0266	0.2387	0.031*	
C6	0.7316 (12)	1.0906 (4)	0.1191 (3)	0.0228 (11)	
H6	0.6182	1.1615	0.1301	0.027*	

### Atomic displacement parameters (Å^2^)

**Table:**

	*U*^11^	*U*^22^	*U*^33^	*U*^12^	*U*^13^	*U*^23^
I1	0.01729 (14)	0.02359 (15)	0.02395 (16)	0.00030 (14)	0.00153 (14)	−0.00153 (15)
Cl1	0.0468 (9)	0.0259 (7)	0.0238 (7)	0.0013 (7)	−0.0056 (6)	0.0049 (6)
N1	0.027 (2)	0.0132 (19)	0.031 (3)	0.003 (2)	−0.001 (3)	0.0002 (18)
C1	0.014 (2)	0.013 (2)	0.029 (3)	−0.002 (2)	−0.003 (2)	−0.001 (2)
C2	0.015 (3)	0.016 (2)	0.019 (3)	−0.0008 (19)	−0.001 (2)	−0.002 (2)
C3	0.018 (2)	0.014 (2)	0.024 (3)	0.0009 (18)	−0.003 (2)	−0.003 (2)
C4	0.025 (3)	0.021 (3)	0.024 (3)	−0.003 (3)	−0.004 (2)	0.002 (2)
C5	0.026 (3)	0.026 (3)	0.025 (3)	−0.003 (2)	0.001 (2)	−0.004 (2)
C6	0.023 (3)	0.015 (2)	0.030 (3)	0.002 (2)	−0.001 (2)	−0.007 (2)

### Geometric parameters (Å, º)

**Table:**

I1—C2	2.101 (5)	C2—C3	1.386 (7)
Cl1—C4	1.755 (6)	C3—C4	1.380 (7)
Cl1—I1^i^	3.7850 (16)	C3—H3	0.95
N1—C1	1.400 (7)	C4—C5	1.387 (8)
N1—H1	0.90 (2)	C5—C6	1.383 (7)
N1—H2	0.90 (2)	C5—H5	0.95
C1—C2	1.395 (7)	C6—H6	0.95
C1—C6	1.402 (7)		
			
Cl1···I1^i^	3.7850 (16)		
			
C4—Cl1—I1^i^	86.03 (18)	C4—C3—H3	120.7
C1—N1—H1	115 (4)	C2—C3—H3	120.7
C1—N1—H2	112 (4)	C3—C4—C5	121.3 (5)
H1—N1—H2	109 (5)	C3—C4—Cl1	119.1 (4)
C2—C1—N1	122.9 (5)	C5—C4—Cl1	119.6 (4)
C2—C1—C6	117.5 (5)	C6—C5—C4	119.2 (5)
N1—C1—C6	119.5 (5)	C6—C5—H5	120.4
C3—C2—C1	122.0 (5)	C4—C5—H5	120.4
C3—C2—I1	117.2 (4)	C5—C6—C1	121.3 (5)
C1—C2—I1	120.8 (4)	C5—C6—H6	119.4
C4—C3—C2	118.7 (5)	C1—C6—H6	119.4
			
N1—C1—C2—C3	176.8 (5)	I1^i^—Cl1—C4—C3	−49.3 (4)
C6—C1—C2—C3	−0.5 (7)	I1^i^—Cl1—C4—C5	129.0 (4)
N1—C1—C2—I1	−3.3 (7)	C3—C4—C5—C6	−0.9 (8)
C6—C1—C2—I1	179.4 (4)	Cl1—C4—C5—C6	−179.2 (4)
C1—C2—C3—C4	0.8 (8)	C4—C5—C6—C1	1.2 (8)
I1—C2—C3—C4	−179.1 (4)	C2—C1—C6—C5	−0.5 (7)
C2—C3—C4—C5	−0.1 (8)	N1—C1—C6—C5	−177.9 (5)
C2—C3—C4—Cl1	178.2 (4)		

Symmetry code: (i) *x*−1/2, −*y*+3/2, −*z*.

### Hydrogen-bond geometry (Å, º)

**Table:**

*D*—H···*A*	*D*—H	H···*A*	*D*···*A*	*D*—H···*A*
N1—H2···N1^ii^	0.90 (2)	2.28 (3)	3.142 (6)	161 (5)

Symmetry code: (ii) *x*−1/2, −*y*+5/2, −*z*.
