# Supplementary figures and images for: Crystal structure of 4-chloro-2-iodo­aniline
Source: Acta Crystallogr Sect E Struct Rep Online. 2014 Aug 1;70(Pt 9):o944–5. doi: 10.1107/S1600536814016869 (PMC4186172; doi:10.1107/S1600536814016869)

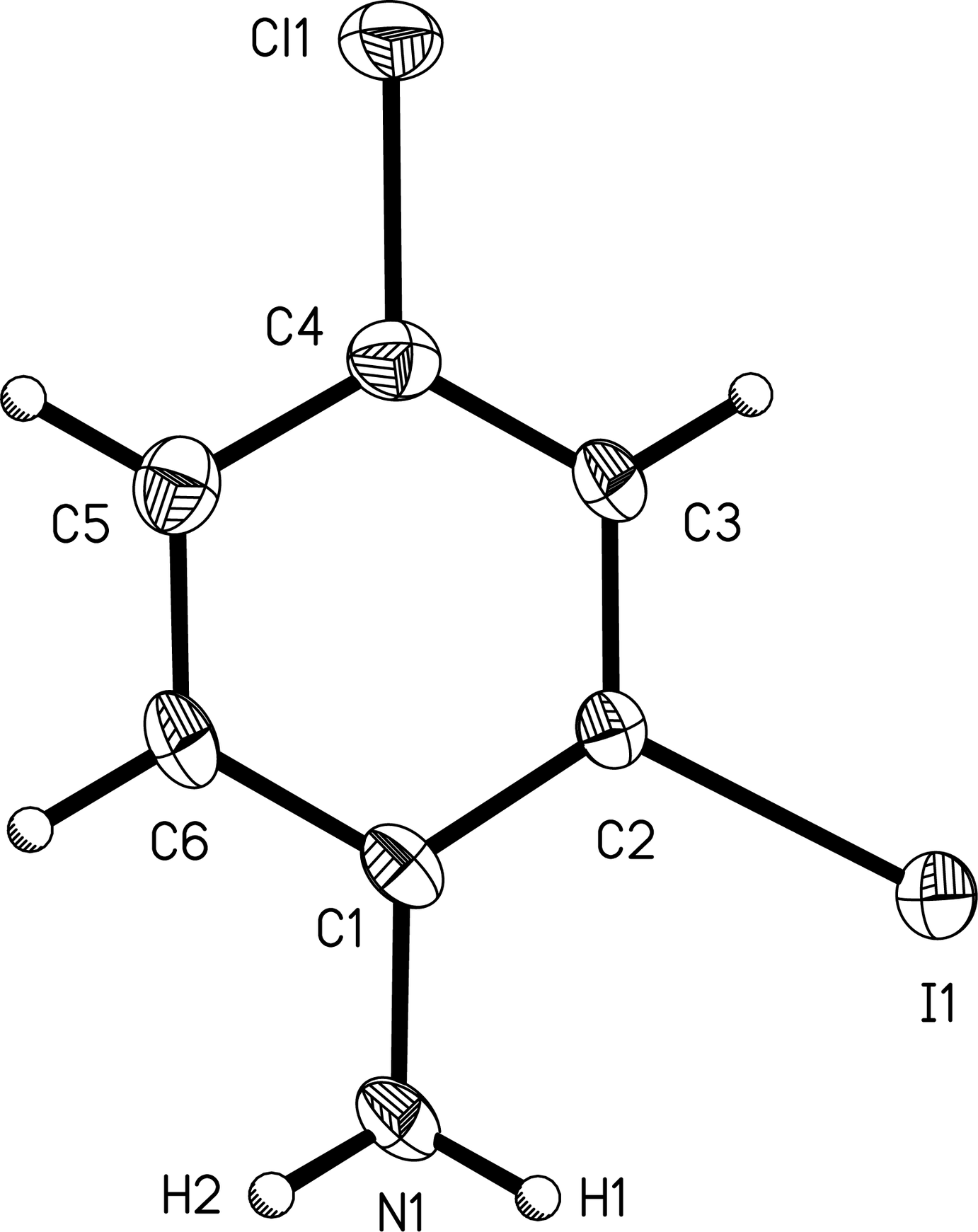

Supplement: Supplementary file 4 [file e-70-0o944-fig1.tif]

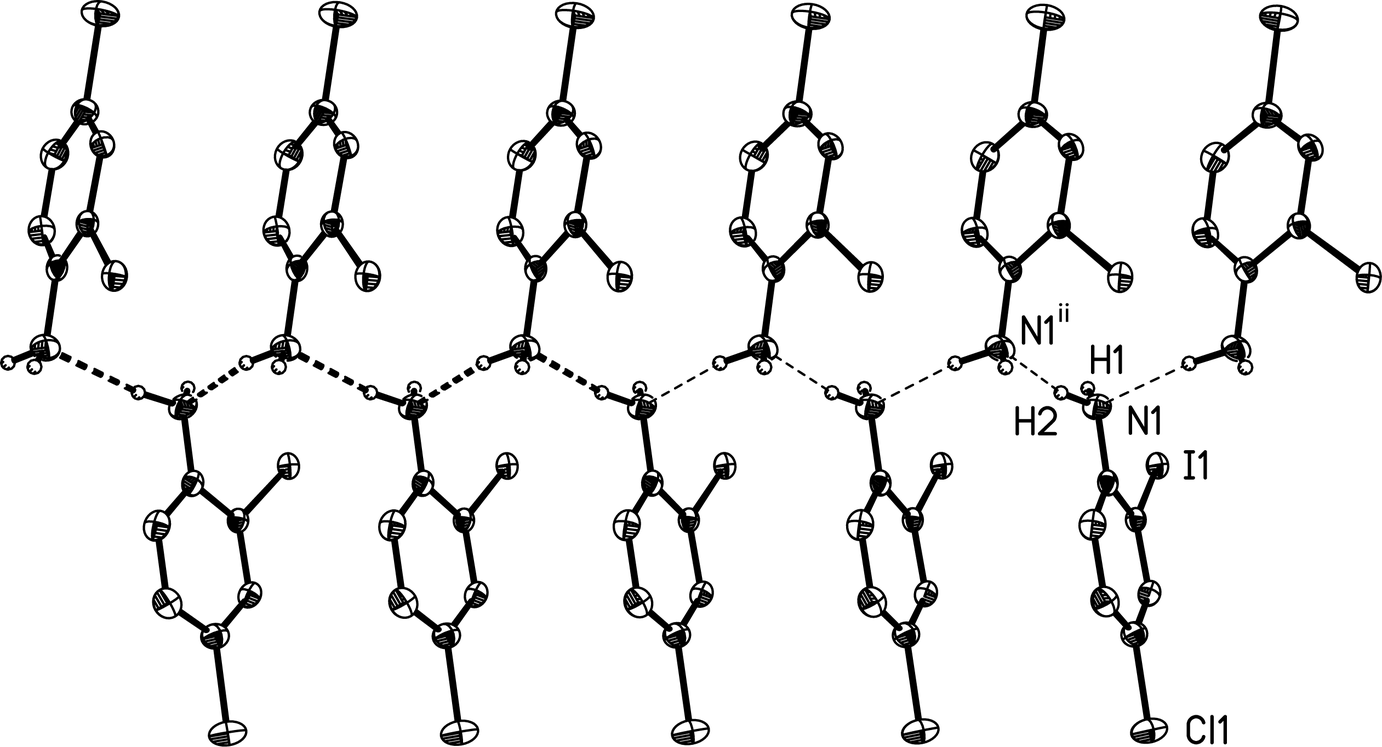

Supplement: Supplementary file 5 [file e-70-0o944-fig2.tif]

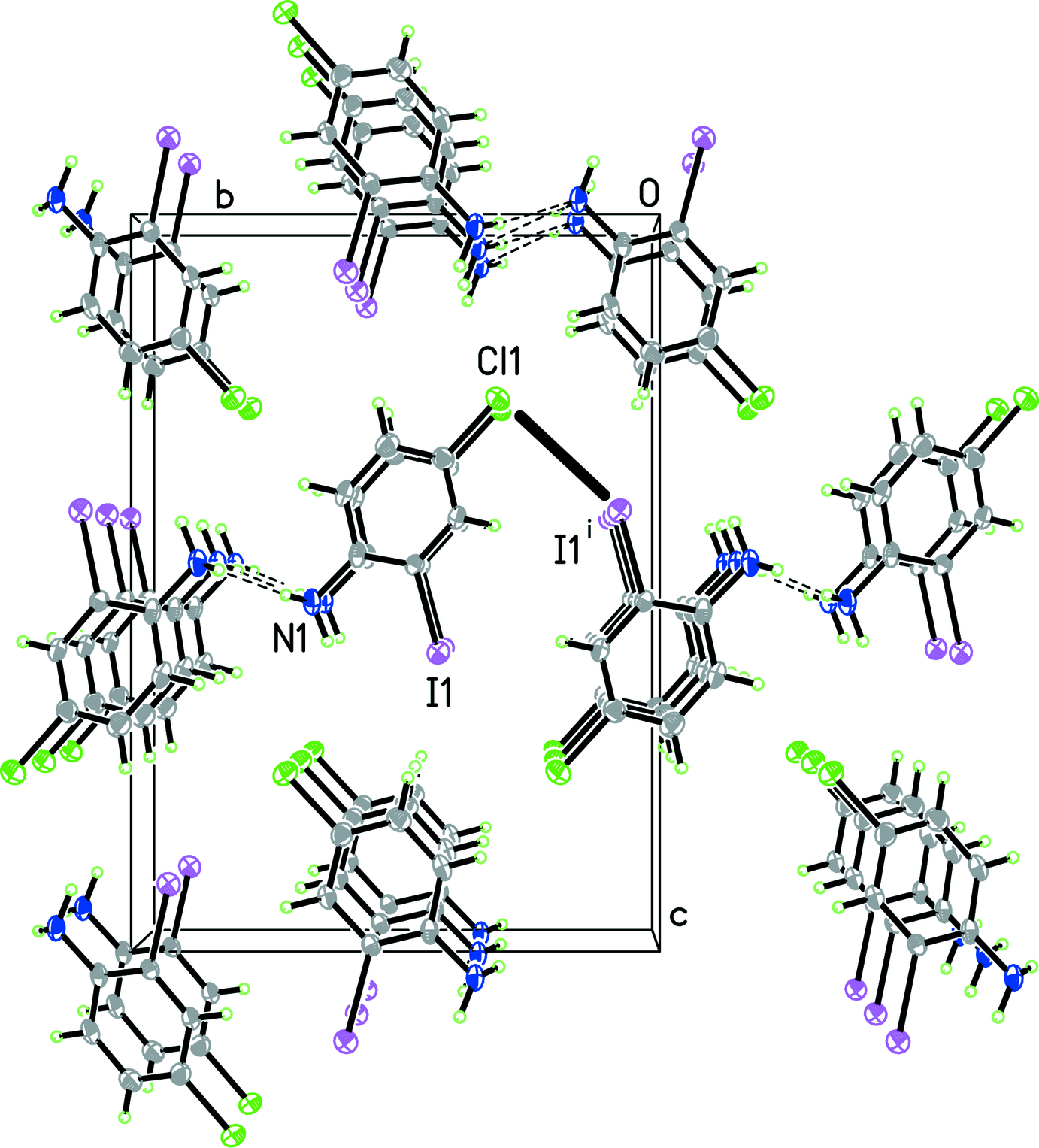

Supplement: Supplementary file 6 [file e-70-0o944-fig3.tif]
